# Nanomaterials for Combating Cancer while Safeguarding Organs: Safe and Effective Integrative Tumor Therapy

**DOI:** 10.34133/bmr.0165

**Published:** 2025-06-12

**Authors:** Keqin Ji, Xianghe Jiang, Zhuanzhuan Zhang, Mengfan Li, Zhu Peng, Yao Wang, Jie Gao

**Affiliations:** ^1^ Changhai Clinical Research Unit, Shanghai Changhai Hospital, Naval Medical University, Shanghai 200433, China.; ^2^ Shanghai Key Laboratory of Nautical Medicine and Translation of Drugs and Medical Devices, Shanghai, China.; ^3^ College of Life Science, Mudanjiang Medical University, Mudanjiang 157011, China.

## Abstract

Cancer remains a leading cause of mortality globally. Combating cancer while safeguarding organs (CCSO) has emerged as a specialized field that employs a multifaceted approach to cancer management. Postsurgery solid tumors face issues such as recurrence and organ dysfunction due to residual cancer, resection, inflammation, and infections. Adjuvant and preventive treatments may also impair organ function, adding to treatment challenges. This review delineates the multifaceted landscape of multidimensional nanomaterials, spanning from 0-dimensional nanoparticles to 3-dimensional scaffolds, and their collaborative roles in concurrent cancer management and organ protection. We underscore the importance of nanomaterial synthesis, functionalization, and responsive release mechanisms in the tumor and organ microenvironments. A comprehensive analysis of nanomaterial applications in integrated cancer management, including melanoma, osteosarcoma, breast cancer, liver cancer, pancreatic cancer, and gastric cancer, is presented, highlighting their potential to overcome therapeutic challenges. The discourse also addresses the obstacles and future directions for nanomaterials for CCSO, offering valuable insights for advancing cancer management and organ protection. This review aims to enhance the comprehension and progress of nanomaterials for CCSO, fostering the development of more effective cancer management modalities.

## Introduction

Cancer remains a predominant cause of mortality globally, posing an important challenge to improving life expectancy. The Global Cancer Statistics 2020 estimate approximately 19.3 million new cancer diagnoses and close to 1 million cancer-related fatalities on a worldwide scale. Projections indicate a potential increase in the global cancer burden to 28.4 million cases by 2040 [1]. Implementing cancer prevention strategies and improving cancer care are imperative for effectively addressing this global health crisis. Holistic integrative oncology (HIO) employs a multifaceted approach to cancer management. HIO merges cutting-edge cancer knowledge with clinical expertise from diverse medical domains. This comprehensive strategy considers the complex interplay of patient-specific factors, including social, environmental, and psychological factors, to craft an individualized cancer treatment regimen [2]. The objective of HIO is to develop a comprehensive and individualized treatment protocol that meets the specific requirements of patients with cancer, aiming to increase treatment efficacy and quality of life [3]. Solid tumors account for 90% of all malignant tumors, and surgical resection is a common treatment for solid tumors; however, solid tumors still face the challenges of metastasis, recurrence, and impaired organ function after surgery for the following reasons: (a) Tumor metastasis and recurrence: Surgical resection may leave behind residual tumor tissues, leading to cancer recurrence and metastasis [4,5]. Surgery may also stimulate the release of catecholamines, which, in turn, promotes tumor growth and angiogenesis [6]. Krall et al. [7] demonstrated that the systemic inflammatory response following surgery promotes the outgrowth of distant immune-controlled tumors in mouse cancer models of dormancy. (b) Impaired organ function, bacterial infection, and chronic inflammation: Surgery may lead to tissue defects, increasing the risk of bacterial infection and chronic inflammation, which, in turn, affects tissue regeneration and organ function [8,9]. Although antibiotics are the main choice for bacterial infections, bacterial resistance is becoming more prevalent. (c) Adjuvant therapies, such as chemotherapy, immunotherapy, and targeted therapies, can cause imbalances in organ homeostasis by disrupting immune homeostasis, physicochemical homeostasis, and metabolic homeostasis, which can damage the functions of organs [10]. Combating cancer while safeguarding organs (CCSO), as one promising strategy in HIO, represents a comprehensive approach that integrates advanced knowledge and practical experience from cancer prevention and treatment [11]. In CCSO, preserving organ functionality is paramount to increasing the quality of life of patients. By reducing iatrogenic harm to organs throughout the therapeutic journey, the adverse effects of treatment can be preempted or alleviated, thus facilitating the preservation or recovery of the patient’s physiological integrity. Therefore, it is essential to optimize cancer prevention and treatment strategies and safeguard organ functions in CCSO [11]. Compared with general nanomaterial-based anticancer therapies, nanomaterials for CCSO have their own characteristics, which makes them perform well in anticancer therapy. For example, nanomaterials for CCSO have highly efficient drug loading and targeting capabilities, which enable precise delivery of anticancer drugs to the tumor site. In addition, nanomaterials for CCSO can take advantage of the differences in physicochemical products between the microenvironment of tumors and normal tissues to exert synergistic antitumor and prorepair effects. In addition, nanomaterials for CCSO emphasize the importance of organ protection, and the core of the CCSO strategy is to minimize damage to normal organs while effectively killing cancer cells. Compared with general anticancer therapies with nanomaterials, nanomaterials for CCSO, by combining factors such as tumor killing factors and tissue repair factors, aim to achieve tumor inhibition while promoting the repair and regeneration of damaged tissues through the efficient delivery and rational release of antitumor drugs and tissue repair factors. In summary, nanomaterials for CCSO have unique advantages in the selection and application of nanomaterials, which can effectively improve the precision and safety of anticancer therapy.

Recent progress in nanomaterial science has catalyzed a transformative impact on CCSO [12,13]. Leveraging these advancements, investigators are now capable of engineering nanomaterials with tailored functions and attributes [14] to realize both cancer management and organ protection in CCSO [15–17]. Nanomaterials can be classified on dimensionality, including 0-dimensional (0D), 1-dimensional (1D), 2-dimensional (2D), and 3-dimensional (3D) constructs. 0D nanomaterials are characterized by nanoscale dimensions in all 3 axes [18]. Nanoparticles and nanopowders, as examples of 0D nanomaterials [19,20], exhibit nanoscale dimensions in 2 axes, with fibrous proteins, carbon nanotubes, and nanowires being examples [11,19]. 2D nanomaterials, characterized by nanoscale thickness along one axis, manifest as thin sheets or membranes [21], with graphene and transition metal dichalcogenides being examples. 3D nanomaterials, which lack nanoscale dimensions, feature complex structures that enable the creation of intricate constructs and tissue mimics, with hydrogels, microspheres, and 3D-printed materials being examples [11]. The development of nanomaterials for CCSO that are adept at integrating anticancer properties with the protection of organ functions is urgently needed. The nanomaterials should be capable of concurrently inhibiting cancer progression and safeguarding the functions of tumor-affected organs. This dual functionality is engineered to prevent the recurrence and metastasis of tumors while concurrently tackling inflammation and infection to facilitate the repair of damaged organs. Nanomaterials for CCSO could significantly increase the potency of therapeutic approaches and, in parallel, increase the living standards of patients.

Nanomaterials for CCSO represent a cutting-edge approach in oncology that synergizes with tumor control agents, tissue repair promoters, diagnostic agents, and other therapeutic modalities. The nanomaterials could also modulate the tumor microenvironment by influencing immune cells and cytokines, stimulating the proliferation and differentiation of local immune cells, and bolstering the immune response against the tumor [22]. This strategy not only eradicates tumor cells but also triggers a cascade of antitumor immune responses that prevent the formation of new tumor foci and metastasis [23,24]. In the context of tissue repair, these nanomaterials could exhibit superior biocompatibility, facilitating the restoration of damaged tissues and protection of organs [25]. By releasing bioactive factors such as growth factors or providing a conducive microenvironment [26,27], these nanomaterials can stimulate tissue regeneration and repair. Their physicochemical properties, including mechanical strength and degradability, are finely tuned to support tissue repair by promoting cell proliferation and angiogenesis, leading to effective restoration and regeneration. Therefore, nanomaterials for CCSO in cancer management and organ protection (short for “nanomaterials for CCSO”) could exert synergistic, convenient, and safe properties, outperforming nanomaterials for cancer management or organ protection [12,13,28]. First, owing to their superior antitumor effects, nanomaterials for CCSO could mitigate tissue damage, enhance tissue repair, and improve treatment tolerance. Second, nanomaterials for CCSO could simplify treatment protocols and reduce patient burden while minimizing the risks associated with exogenous materials and reducing toxicity. Despite these advancements, the literature on nanomaterials for CCSO is still limited, with only a few reviews on this emerging subject [11,13,29]. These reviews summarize the rational construction of multifunctional biomaterials with intrinsic therapeutic properties and tissue regenerative bioactivity on the basis of the intrinsic response to external physical triggers (e.g., photon response or magnetic field response) or endogenous disease microenvironments (e.g., weak acidity or excess hydrogen peroxide), as well as tissue regenerative bioactivities and applications in integrated regenerative therapies, including tissue regenerative–tumor therapy, tissue regenerative–infection therapy, tissue regeneration–tumor therapy, tissue regeneration–infection therapy, and tissue regeneration–inflammation therapy. Furthermore, our group also conducted several reviews on nanomaterials for integrated therapy of osteosarcoma and melanoma [30–32]. However, all these reviews focus on only one or several categories of nanomaterials for CCSO, and the discussed cancers mainly include osteosarcoma and melanoma [30–32]. Our review summarizes the application of nanomaterials for CCSO with different dimensions and functionalities in the integrated therapy and prevention of melanoma, osteosarcoma, hepatocellular carcinoma, and breast, pancreatic, and gastric cancer. Therefore, it is imperative to comprehensively review the design principles, mechanisms, and applications of nanomaterials for CCSO, and this review will provide new insights and new directions for CCSO.

**Fig. 1.**
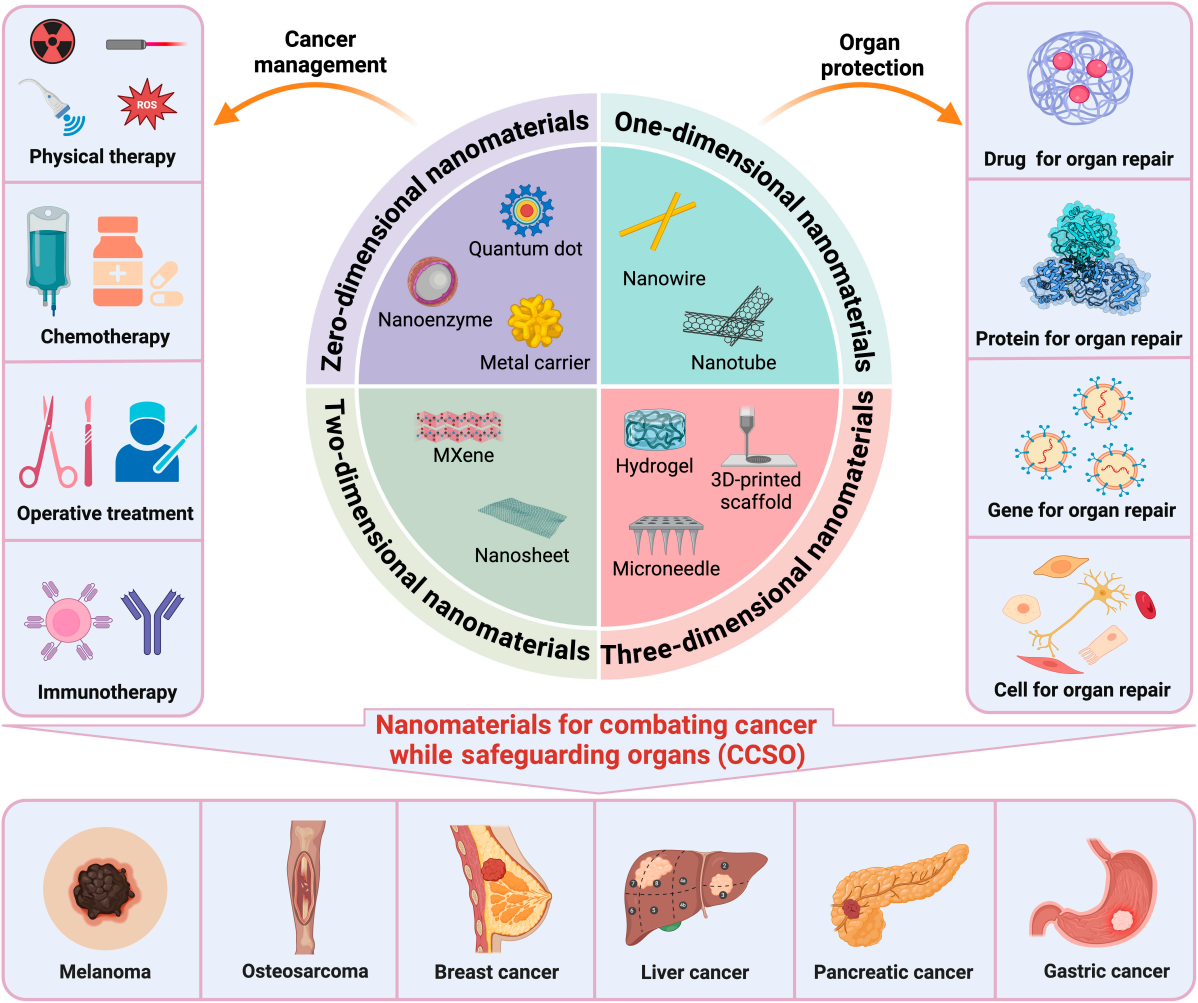
Schematic diagram of the classification and utilization of nanomaterials for CCSO. These nanomaterials are systematically categorized into 0D, 1D, 2D, and 3D materials. They exert therapeutic effects on tumors through physical therapy, chemotherapy, surgery, and immunotherapy and are also adept at facilitating organ protection by delivering drugs, proteins, genes, and cells. Their applications are crucial for managing cancer and protecting organs across a variety of cancers, including melanoma, osteosarcoma, breast cancer, liver cancer, and pancreatic cancer. The images were created with BioRender.

This review provides a thorough examination of the current research landmarks and practical applications of nanomaterials for CCSO. This review systematically reviews the use of nanomaterials for CCSO in a variety of cancers, including melanoma, osteosarcoma, liver cancer, pancreatic cancer, and gastric cancer (Fig. 1). Finally, we discuss the challenges, limitations, and future directions of nanomaterials for CCSO, emphasizing their significant potential for clinical application.

## Classification of Nanomaterials for CCSO

There are various classification methods for nanomaterials in CCSO. Based on their chemical composition, they can be categorized into metals, ceramics, polymers, and composite materials [19]. Additionally, based on the interaction between nanomaterials and the human body, they can be classified into biocompatible materials, bioactive materials, and biodegradable materials [33]. On the other hand, based on the origin of nanomaterials, they can be divided into natural nanomaterials and synthetic nanomaterials. Synthetic nanomaterials include synthetic polymers and bioactive glasses, etc. [34]. Natural nanomaterials include proteins, polysaccharides, and collagen, which are present in biological organisms and exhibit excellent biocompatibility and bioactivity[35,36]. In fact, the geometrical shapes of nanomaterials play a key role in determining the behaviors of cells [19].

### Small-molecule drugs

A variety of small-molecule drugs exhibit both antitumor and prorepair capabilities. Phenolic compounds such as epinephrine sulfate and dopamine could possess antitumor capabilities and enhance tissue repair [37,38]; antioxidants, including glutatCCSOne and vitamin E, exhibit antitumor effects, reduce oxidative damage, and foster tissue regeneration; estrogenic drugs, such as estradiol, aid nerve cell repair and have antitumor properties; methyldopa, an antihypertensive agent, also has antitumor activity and supports tissue repair in organs such as the kidneys and liver; and polyphenolic compounds, notably curcumin and tannic acid, are extensively used to combat cancer and support tissue regeneration. Curcumin can not only target various stages of cancer but also mitigate inflammation, neutralize reactive oxygen species (ROS), and stimulate growth factors to promote tissue repair [27,39,40]. Tannic acid, a naturally occurring polyphenol, possesses dual therapeutic potential. It augments the healing process through its antioxidant, anti-inflammatory, and collagen-stimulating properties while simultaneously hindering tumor progression through the inhibition of cell proliferation, induction of apoptosis, and modulation of immune responses [41,42].

### 0D nanomaterials

0D nanomaterials usually refer to particles or structures with nanometer-scale dimensions that are very small in all 3 spatial dimensions, usually on the order of a few nanometers to tens of nanometers [43]. 0D nanomaterials usually have a high specific surface area and special physical and chemical properties and therefore have a wide range of applications in drug delivery, bioimaging, and biosensing. Owing to their small size and special surface properties, 0D nanomaterials have many advantages, such as good cell permeability, tunable drug release behavior, and high drug loading and targeting [43]. Inorganic nanomaterials are composed mainly of inorganic nonmetallic materials, including metals such as various metal monomers, sulfides, oxides, or silicides. Organic nanomaterials include liposomes, proteins, polysaccharide nanomaterials, and so on. Tumor killing can be achieved not only by delivering drugs but also by generating heat and ROS under the action of a magnetic field, light, microwave, or ultrasound [44–46]. For example, inorganic nanomaterials such as nanoscale black titanium dioxide (B-TiO_2−*x*_) can achieve potent tumor-killing effects through both photothermal effects and ROS generation when exposed to a single wavelength of near-infrared (NIR) radiation [21]. In addition, ROS not only kill tumor cells, but also play a role in antimicrobial and promoting tissue repair. For example, oxygen vacancies on the exposed surface of ZnO nanosheets exhibit excellent antibacterial activity in the dark [47]. A study reported a silicone-based nanomedicine (ZnPP@FQOS) that not only kills tumor cells but also remodels tumor-associated fibroblasts in the tumor microenvironment [48]. This dual action makes ROS a promising therapeutic strategy for both cancer treatment and organ protection.

In the realm of 0D nanomaterials, the dual capabilities of tumor ablation and tissue repair can be realized if the nanomaterial exhibits specific properties, such as photothermal and photodynamic properties, to eradicate tumors, coupled with enzymatic activity to neutralize ROS. A plethora of such nanomaterials, including copper sulfide, Prussian blue, and molybdenum disulfide, have been identified [49,50]. Our group reported a tumor cell membrane-encapsulated Prussian blue nanozymes (PBVac). Postoperative melanoma patients face the challenges of difficult-to-heal wounds, tumor remnants, infections, and radiation dermatitis, which seriously affect patient prognosis. We utilized the photothermal, antimicrobial, and antioxidant effects of PBVac nanoenzymes to achieve photothermal antitumor effects, as well as to treat wounds and radiation dermatitis by stimulating angiogenesis and exerting antioxidant and anti-infective effects; for the first time, this study provides a systemic solution for the comprehensive treatment of postoperative complications of melanoma [51]. In addition to metal nanoenzymes, researchers have used plant extracts with reducing properties to reduce metals and have prepared them as plant extracts/metal nanomaterials for CCSO. For example, silver nanoparticles (LTAgNPs) are formed by the reduction of *Linyphia triangularis* hemiptera, and these nanomaterials exhibit excellent antimicrobial, antioxidant, and antitumor activities and promote wound healing [52]. Silver nanoparticles (AgNPs) are also generated by secondary metabolites of *Rhizopus aculeatus* and have similar functions [53]. Metal oxides, which have excellent photothermal, magnetothermal, and other functionalities, have also been widely used in cancer management and organ protection. However, metal oxides are often composited with 3D nanomaterials; for example, Wang et al. constructed nanoscale B-TiO_2−*x*_ nanocrystals loaded with a chitosan matrix and developed structural defects in TiO_2_ nanocrystals by fabricating them in TiO_2_ nanocrystals. B-TiO_2−*x*_ has photothermal effects and generates ROS under NIR irradiation. By further integrating B-TiO_2−*x*_ into a light-injectable chitosan (CTS) matrix, the photothermal therapy (PTT)/photodynamic therapy (PDT) effect generated by this thermogel significantly inhibited skin tumor growth, whereas the release of Mg^2+^ ions from Mg-containing B-TiO_2−*x*_ nanoparticles accelerated the healing of chronic wounds in mice [54].

### 1D nanomaterials for CCSO

1D nanomaterials typically refer to fibrous, linear, or nanotube-like structures that are highly controllable and tunable, offering potential applications in antitumor and prorepair scenarios [55]. For example, nanocellulose, a biodegradable material composed of cellulose nanocrystals, exhibits high biocompatibility and biodegradability, mimicking the microstructure of the extracellular matrix [56]. Nanocellulose can serve as a drug carrier for the release of antitumor medications and can be utilized in tissue engineering and wound repair to increase cell adhesion and growth [57]. Collagen, a protein predominantly found in human tissues, is both biocompatible and biodegradable [58]. Collagen nanofibers can be fabricated via techniques such as electrospinning or self-assembly, mimicking the nanostructure of collagen fibers, providing scaffolding and directional cues for cells, and potentially stimulating cell migration and repair [59,60]. These 1D nanomaterials are currently being investigated for antitumor and prorepair purposes, and their design and application are anticipated to offer innovative solutions in the realms of tumor therapy and tissue engineering.

### 2D nanomaterials for CCSO

2D nanomaterials, defined by their nanoscale thickness of at least one dimension, exhibit a marked reduction in size perpendicular to their surfaces, resulting in ultrathin structures with unique physical, chemical, and electronic properties compared with those of traditional 3D nanomaterials [61,62].

Black phosphorus nanosheets (BPNSs), as representative 2D nanomaterials, have potent antitumor effects and promote tissue repair. The oxidation of BPNSs enhances the release of phosphate, which reacts with calcium ions to expedite the formation of calcium phosphate (CaP), thereby facilitating bone repair [63]. After implantation into a mouse model of osteosarcoma, black phosphorus–bioactive glass (BP–BG) composite scaffolds quickly increased the temperature, effectively suppressed tumorigenesis, and demonstrated superior bone defect repair capabilities compared with those of pure BG scaffolds [63]. The composite scaffold possesses significant antimelanoma activity and wound healing-promoting effects and is a novel therapeutic approach for enhancing postoperative wound management in melanoma treatment [64].

The integration of 0D with 2D nanomaterials leverages their synergistic properties to achieve both antitumor efficacy and pro-bone repair, which is pivotal for CCSO. To fabricate a stable and cost-effective photothermal film for bone implants, Yao et al. developed a Ni nanoparticle-doped oxide semiconductor film on a nickel–titanium alloy by reducing Ni–Ti layered double hydroxides. This film demonstrated potent antitumor capabilities for ex vivo PTT. Moreover, the Ni nanoparticles embedded in the film facilitated bone differentiation, offering a novel multifunctional Ni–Ti bone implant design as a promising strategy for addressing bone tumor-associated defects [65]. Parallel to this approach, the design of nanomaterials endowed with photothermal properties can be integrated with 2D materials to enhance repair effects. Geng et al. created a novel, low-toxicity bifunctional 0D/2D heterojunction (HJ) that harnesses the photothermal effect in the second NIR window (NIR-II) for concurrent osteosarcoma treatment and bone regeneration. Additionally, the mild photothermal effect of HJ notably promoted osteogenic differentiation and the up-regulation of bone-related genes [66].

2D nanomaterials are burgeoning agents with dual potential in antitumor treatment and repair promotion. First, as drug delivery vectors, 2D nanomaterials exploit their nanoscale dimensions and expansive surface area to facilitate efficient drug loading and targeted delivery, with surface modifications enhancing tumor cell specificity and therapeutic efficacy [67]. Second, PTT is achievable with certain 2D materials, such as molybdenum disulfide and BP, which are light sensitive and can convert optical energy into heat for precise tumor ablation with minimal damage to surrounding tissue [68]. Third, these nanomaterials can modulate the immune system to bolster immunotherapy by utilizing their photothermal properties to trigger tumor cell apoptosis and antigen release, thereby stimulating an immune response [69]. In the realm of repair, 2D nanomaterials, such as graphene and molybdenum disulfide, demonstrate promise in bone repair by stimulating osteoblast proliferation and bone matrix synthesis, thus assisting in fracture healing and defect repair. Additionally, they can enhance wound healing by providing structural support to the extracellular matrix and regulating cellular signaling to increase angiogenesis and cell migration. Furthermore, they exhibit potential in nerve repair by supporting nerve cell growth and regeneration, offering directional cues for nerve regrowth. While 2D nanomaterials have broad application prospects in oncology and regenerative medicine, ongoing research and clinical trials are essential to confirm their safety and efficacy and mitigate any potential toxicity or biocompatibility concerns.

### 3D nanomaterials for CCSO

#### 3D nanomaterials

3D nanomaterials are intricately structured nanomaterials designed to support and guide tissue growth and regeneration. Composed of biodegradable or biologically inert materials, they feature a porous architecture and tailored mechanical properties [70]. These scaffolds are instrumental in tissue engineering and regenerative medicine, offering a conducive environment for cell attachment, proliferation, and differentiation, thereby facilitating tissue repair and regeneration. In antitumor therapy, 3D scaffolds provide benefits such as high drug loading capacity, localized drug delivery, combination treatment strategies, and the capability for imaging and monitoring [29,70]. As stable drug carriers, 3D scaffolds mitigate early drug release and degradation, enhancing drug stability and extending release duration [71,72]. Furthermore, by incorporating specific ligands or antibodies on their surface, these scaffolds can achieve tumor cell-specific recognition and targeted drug delivery, enhancing therapeutic efficacy while minimizing toxic side effects [64,73]. Additionally, 3D scaffolds can be loaded with multiple antitumor agents and therapeutic substances to enable combined therapy, combat drug resistance, and eliminate the need for multiple administrations [74]. For example, doxorubicin (DOX), an anticancer drug, was incorporated into porous scaffolds alongside copper nanoparticles, with photothermal heating accelerating DOX release to synergize chemotherapy and PTT for melanoma treatment [75].

Hydrogels, 3D nanomaterials, are extensively utilized in integrated cancer therapy. Huang et al. [76] demonstrated their efficacy in melanoma growth inhibition, bacterial infection treatment, and wound healing by incorporating Ag_2_S nanodots conjugated with Fe-doped BG nanoparticles (BGN-Fe-Ag2S) into biodegradable polyethylene glycol diacrylate (PEGDA) and 2,2ʹ-azobis[2-(2-imidazolin-2-yl)propane]dihydrochloride (AIPH) solutions. He and Li [77] synthesized a bifunctional hydrogel system by grafting the connexin43 (Cx43) mimetic peptide juxtamembrane 2 (JM2) onto hyaluronic acid (HA-JM2), which serves as a carrier. This injectable hydrogel concurrently inhibited tumor recurrence and promoted wound healing. JM2 was found to suppress tumor cell proliferation and induce apoptosis in vitro, effectively inhibiting tumor growth in vivo. Sustained JM2 release from the HA-JM2 hydrogel aids in controlling the inflammatory response and stimulating angiogenesis, thereby accelerating wound healing in total skin defects. Overall, the HA-JM2 injectable hydrogel has significant potential for postoperative tumor therapy applications. Zhang et al. [78] prepared magnetic bioactive microcrystalline glass (MBGC) with both bioactivity and thermotherapeutic capabilities by leveraging magnetic Fe_3_O_4_ within a (CaO-SiO_2_-P_2_O_5_-MgO) scaffold. MBGCs exhibit favorable magnetic and magnetothermal properties suitable for tumor resection and stimulate osteoblast proliferation, alkaline phosphatase activity, and osteogenic expression, making them promising candidates for bone tumor treatment and bone defect regeneration.

Another popular 3D nanomaterial is bioceramic scaffolds. Huang et al. prepared black (B-AKT) bioceramic scaffolds with micro/nanostructures that can activate the bone morphogenetic protein signaling pathway to initiate the osteogenic differentiation of bone marrow mesenchymal stem cells (MSCs) and further stimulate in vivo bone formation. Furthermore, the produced B-AKT scaffolds exhibited good photothermal and anticancer effects when exposed to NIR radiation (0.66 W·cm^−2^) at temperatures higher than 100 °C [79]. 3D nanomaterials are frequently produced via 3D printing technology. Long et al. employed 3D printing to create a multifunctional magnesium-bonded scaffold for the full postoperative care of osteosarcoma patients. Additionally, 3D-printed BG scaffolds (MBS) and 2C MXene encapsulated with S-nitrosotCCSOl (R-SNO)-grafted mesoporous silica have been combined to create nanomaterials [80].

#### 3D hybridization with 0D nanomaterials

The long-lasting gradual release of 0D nanomaterials can be achieved by combining them with 3D hydrogels to create 3D hybrids with 0D nanomaterials that have prorepair and antitumor properties. Several investigations have combined organic and inorganic nanoparticles with scaffold materials to create both prorepair and antitumor hydrogels. One popular approach is the hybridization of hydrogels with inorganic nanoparticles for prorepair and antitumor tumor integration. Metal monoliths [21,81], oxymetallic carriers [76,82,83], sulfide metal carriers [73,74,84], and silicide carriers are examples of common inorganic nanomaterials [85,86].

Metal oxides possessing exceptional photothermal, magnetothermal, and other functionalities have also been extensively applied in the fields of tumor suppression and tumor integration to promote repair. One example is the B-TiO_2−*x*_ nanocrystals previously discussed. Additionally, investigations have been conducted on the fabrication of mesoporous bioglass (BG)/chitosan (CS) porous scaffolds (MBCSs) modified with magnetic SrFe_12_O_19_ nanoparticles. The MBCS-induced magnetic field increased the expression of osteogenesis-related genes, including osteocalcin, collagen type 1, recombinant runt-related transcription factor 2, and alkaline phosphatase, promoting new bone regeneration. Furthermore, the use of nanoparticles in MBCSs increased the efficiency of photothermal conversion. When exposed to NIR laser light, the increased temperature of tumors that were cocultured with MBCSs caused apoptosis and ablation [87]. Fe_3_O_4_ nanoparticles exhibit an enhanced ability to absorb NIR light and convert it into heat more efficiently. Researchers have introduced Fe_3_O_4_ nanoparticles into a porous gel (Gel/Fe_3_O_4_) to enable the gel to facilitate tumor ablation via NIR-triggered photothermal conversion. The composite scaffold facilitated cell adhesion and proliferation, whereas the released Fe^3+^ promoted the expression of bone-related genes, hence stimulating tissue regeneration. This highlights the significant potential of the scaffold for tissue regeneration [88]. In addition to including one type of metal oxide in hydrogel scaffolds, it is possible to incorporate 2 types of metal oxides that possess the capabilities of PTT for tumors and tissue regeneration into hydrogel scaffolds. This allows for both tumor therapy and tissue regeneration.

Metal sulfides, known for their exceptional photothermal and magnetothermal properties, are extensively employed in the fields of tumor treatment and tissue regeneration. Oncologic surgery often leads to the presence of tumor remnants, tissue deficiencies, and infections caused by bacteria that are resistant to several drugs. As a consequence, there is a significant likelihood of tumor recurrence, low survival rates, and the development of long-lasting wounds. This work aimed to create a photoactivatable injectable hydrogel by incorporating BGN-Fe-Ag_2_S nanoparticles into biodegradable PEGDA and AIPH solutions. The hydrogel was designed to limit tumor growth, cure bacterial infections, and promote wound healing. These radicals then initiate the gelation of PEGDA. The in situ gelation hydrogel formed by incorporating iron into BGN-Fe-Ag_2_S had remarkable photothermal and chemodynamic properties. This hydrogel effectively eradicated multidrug-resistant (MDR) bacteria and efficiently destroyed tumors during the therapeutic procedure. Furthermore, the hydrogel effectively accelerated the process of wound healing and enhanced the growth of skin appendages in a model of full-thickness skin wounds, mostly as a result of the hydrolysis of BG. These findings indicate that this versatile hydrogel is an appropriate biomaterial for suppressing tumor growth and combating bacterial infections in tissues following surgical tumor excision [76]. PTT shows great potential as a cancer treatment method. However, this process is hindered by the occurrence of localized damage and the subsequent development of bacterial infections. To address these difficulties, researchers have explored techniques for implementing mild PTT following hyperthermal tumor ablation. The aim is to produce synergistic outcomes of tumor suppression, wound healing, and bacterial eradication via the use of hydrogels. This work utilized Bi_2_S_3_ nanorods as photothermal agents and applied a coating of hyaluronic acid to achieve BiH nanorods with excellent colloidal stability. This hydrogel has the potential to be utilized for targeted cancer therapy via high-temperature PTT, followed by wound healing through mild hyperthermia and the stimulation of cell proliferation via allantoin. Furthermore, a remarkable ability to prevent bleeding was observed as a result of the water absorption capacity and negative charge of bitter almond gum and sodium alginate, along with the porous structure of the hydrogel. The hydrogel effectively eliminated infections via localized heat generation and the natural antibacterial activity of the nanorods. The photothermal heat generated by hydrogels has been demonstrated to effectively eliminate tumors and enhance wound healing in in vivo investigations [73].

Metal silicides have been extensively employed for both anticancer and prorepair tumor integration purposes. Ma et al. fabricated composite sodium alginate hydrogels containing β-FeSi_2_ nanoparticles, which enhance the healing of skin wounds caused by tumors and inhibit cancer through photothermal and chemokinetic treatments. The iron and silicon ions that are produced enhance the movement and specialization of endothelial cells, as well as the formation of new blood vessels in skin wounds. Therefore, this sprayable hydrogel has the benefit of being easily transported for urgent wound care. To summarize, the composite hydrogel, formed by incorporating FS into the sprayable β-FeSi_2_-incorporated sodium alginate (FS/SA) hydrogel, possesses both tumor treatment and skin wound healing capabilities [85].

Organic nanomaterials possess favorable biocompatibility and minimal toxicity. Dopamine, a representative organic substance, has remarkable photothermal properties and promotes bone formation. It is commonly employed in tumor therapy and tissue regeneration to integrate and control nanomaterials. SA-GG@PDA-hybridized hydrogel scaffolds coated with a DOX thermogel effectively suppressed melanoma recurrence and promoted wound healing via sequential PTT and chemotherapy [72]. The chemotherapeutic medication DOX is encapsulated within a thermal gelatin hydrogel. This scaffold facilitates the rapid release of the drug when exposed to photothermal triggering. The objective is to achieve photothermal chemotherapy, which effectively inhibits the proliferation and recurrence of tumor cells. The porous hybridized hydrogel scaffold facilitated wound healing and increased the proliferation and migration of vascular endothelial cells. Additionally, this nonhomogeneous SA-GG@PDA + DOX hydrogel scaffold inhibited tumor recurrence and improved wound healing after surgery [89].

Graphene oxide, like polydopamine (PDA), is a frequently used organic nanomaterial that has a photothermal effect. Ge et al. fabricated a composite chitosan scaffold by including graphene oxide (GO) nanoparticles and CePO_4_ nanorods. This scaffold was specifically designed for the treatment of bone metastases in breast cancer patients [90].

#### 3D hybridization with 1D or 2D nanomaterials

The combination of 3D hydrogels with 1D or 2D nanomaterials can also generate tumor hydrogel materials that integrate both antitumor and prorepair effects. Shanmugapriya et al. developed carboxymethyl cellulose nanofibers that included fucoidan/alginate-based junction cold gel for the purpose of creating a hydrogel that promotes wound healing. Hydrogels can eliminate cancer cells by increasing the quantity of ROS derived from univalent oxygen. The acceleration of wound healing was attributed to the increased presence of fibroblasts and increased expression of collagen fibers, which are known for their high protein content. Therefore, these findings indicate that hydrogels are potent therapeutic agents that can be utilized to facilitate efficient and expeditious healing of skin wounds [91].

Composite hydrogels consisting of 2D nanoparticles can also generate integrated cancer hydrogel materials with both antitumor and prorepair properties. The reappearance of skin cancers following surgical removal and the healing of wounds caused by MDR bacterial infections pose significant challenges in both clinical practice and research. Wang et al. created a bioexcited MnO_2_ hydrogel called bio-inspired MnO_2_ hybrid (BMH) that is injectable and responsive to both redox and light. This hydrogel is useful for treating melanoma via photothermal chemotherapy and promoting wound healing in bacterial infections that are resistant to multiple drugs (MDRs) [92]. They proved that their hydrogel was highly efficient in nearly completely inhibiting the growth of large solid tumors both under laboratory conditions and in living organisms. Furthermore, BMH effectively enhances wound healing in living organisms, particularly in cases of MDR infections. BMH hydrogels exhibit significant therapeutic potential in the fields of cancer therapy and tissue engineering. Table 1 provides a summary of multidimensional nanomaterials that have both antitumor and prorepair properties.

**Table 1.** The antitumor and prorepair effects of multidimensional nanomaterials for CCSO

Names	Characteristic	Types	Antitumor and prorepair effects	References
Integrated small-molecule drugs	Small-molecule drugs are usually combined with composite scaffold materials to achieve controlled release of drugs through the encapsulation of small-molecule drugs by nano-scaffold materials to exert antitumor and prorepair effects.	Biliverdin	Biliverdin is often combined with hydrogels to achieve slow release and prolonged cholecalciferol action, thereby killing cancer cells in vitro and inhibiting tumor growth in vivo.	[36]
		Vitamin C and vitamin E	Neutralizes free radicals, reduces oxidative stress damage, protects cells and tissues from oxidative damage, and promotes tissue repair	[157]
		Epinephrine and dopamine	Possesses antitumor activity and promotes tissue repair and regeneration	[133,137]
Zero-dimensional nanomaterials	Zero-dimensional nanomaterials include inorganic materials and metallic nanocarriers, and zero-dimensional nanocarriers are usually combined with scaffolding materials to achieve tumor killing by utilizing the photothermal properties of the inorganic and metallic materials, while promoting tissue repair through stimulation of angiogenesis and antioxidant protection.	Nanoscale black titanium dioxide (B-TiO_2−*x*_)	It has the dual function of photothermal effect and ROS generation under single-wavelength near-infrared irradiation, which can realize the strong killing of tumors.	[20]
		Polydopamine (PDA)	Excellent photothermal performance under near-infrared triggering, thus demonstrating significant tumor cell killing capability	[20,37]
Two-dimensional nanomaterials	Two-dimensional nanomaterials, mainly including nanofiber scaffolds and nanosheets, usually have good photothermal properties. Two-dimensional nanomaterial composite scaffolds can achieve antitumor and tissue repair by providing the scaffolds with the desired photothermal conversion function under near-infrared laser irradiation.	BP–BG composite bracket	BP nanosheets implanted in surgically resected bone defects were used for photothermal therapy using their excellent photothermal properties, while the inherent physicochemical properties of the BP nanosheets gave them excellent in situ biomineralization properties to promote osseointegration.	[8]
		GP nanofiber scaffold	BPNSs are loaded with the anticancer antibiotic adriamycin (DOX) and coupled with NH2-PEG-FA for tumor-targeted delivery. Most BP-based nanoformulations are used for synergistic photothermal therapy and heat-triggered DOX antitumor therapy, and BPNSs can be gradually degraded to phosphates/phosphonates, which enhances tissue repair through activation of ERK1/2 and PI3K/Akt pathways.	[29]
Three-dimensional nanomaterial	Three-dimensional nanomaterials, including simple scaffold materials and composite scaffold materials, are usually combined with small-molecule drugs as well as multidimensional nanomaterials, which have good antitumor, fibroblast proliferation-promoting, and angiogenesis-promoting effects.	Cur-MP/IR820 loaded methylcellulose hydrogel	Curcumin can be used as a bone repair agent with various functions such as antitumor and osteogenic induction Interestingly, PLGA-based microspheres are long-term stable drug release carriers, so the sustained release of curcumin inhibits residual tumor cells after NIR irradiation and induces osteogenic differentiation of bone marrow mesenchymal stem cells (BMSCs) for bone reconstruction.	[34]

ROS, reactive oxygen species; BP–BG, black phosphorus–bioactive glass, BPNS, black phosphorus nanosheet; PEG, polyethylene glycol; ERK, extracellular signal–regulated kinase; PI3K, phosphatidylinositol 3-kinase; PLGA, poly(lactic-co-glycolic acid); NIR, near-infrared

### Living 3D nanomaterials

Living 3D nanomaterials refer to substances that possess biological properties and capabilities, including algae, MSCs, and bacteria. These substances are crucial for their ability to combat tumors and protect organs [93–96]. Algae include inherent antioxidants and anti-inflammatory compounds that aid in combating cancers and diminishing inflammatory reactions. MSCs can differentiate in multiple directions and have immunomodulatory properties that contribute to the healing and regeneration of tissues. In contrast, bacteria generate antibiotics and other bioactive compounds that combat tumor cells and facilitate the healing of wounds. These bioactive materials show great potential for use in the biomedical industry [97,98].

Algae refer to organisms that can perform photosynthesis but lack embryonic features, as well as nonphotosynthesizing species that closely resemble them. These creatures have a basic structure, and many of them consist of a single cell. They are categorized into 11 phyla: Cyanobacteria, Red Algae, Cryptophyta, Methanogens, Golden Algae, Flavobacteria, Diatoms, Brown Algae, Nudibranches, Chlorophyta, and Verticillasters. Algae can be categorized into microalgae and macroalgae on the basis of their size and into green, brown, and red algae on the basis of their color. Algae can be categorized into eukaryotic and prokaryotic forms [99,100]. Our laboratory has recently published review articles that provide a comprehensive analysis of the antitumor and prorepair mechanisms of *Chlorella* [101,102]*.* Algae can combat malignancies through diverse mechanisms: (a) The hypoxic tumor microenvironment can be enhanced to directly kill and prevent tumor growth [103]. Certain compounds found in certain types of algae, such as chlorophyll and phycocyanin, function as natural photosensitizers. These photosensitizers can be used in specific applications to generate ROS at specific wavelengths, thereby triggering the destruction and death of tumor cells. [104]. Algae can serve as drug delivery vehicles to transport antitumor medication compounds, specifically to tissues or cells [105]. Algae can be used for tumor imaging via autofluorescence and synthetic alterations. Additionally, algae can influence tumor cell lineages and the cell cycle [106]. These pathways significantly amplify the antitumor effects of algae. Indeed, the anticancer activities of these substances frequently necessitate additional chemical or physical alterations to augment their biological efficacy or physicochemical characteristics for therapeutic applications. For example, techniques such as cell membrane encapsulation, hydrogel engineering, and algal engineering modification have been used [107,108]. (b) Algae also have promising potential in facilitating tissue regeneration. Algae possess favorable hydrophilicity and biocompatibility. Certain components found in algae enhance coagulation and vasoconstriction, hence assisting in wound healing and hemostasis. Algae efficiently cover shallow wounds and utilize their distinct biological capabilities to produce oxygen, improve the condition of low oxygen levels in the wound (wound hypoxia), provide a favorable environment for wound repair and healing, and accelerate the healing process [101,109]. Nevertheless, the process of industrializing algae is contingent upon various factors. First, the toxicity of the bioactive compounds found in algae remains uncertain. Additionally, the use of algae for drug delivery may present challenges such as particle aggregation and diminished effectiveness. The toxicity of the active substances in algae is currently unknown. The use of algae for drug delivery may involve issues such as particle aggregation and decreased effectiveness. Additionally, there are challenges that need to be resolved regarding the risk of infection, inflammation, and immune response in the case of synergistic PDT. Additional investigations and innovations are needed to fully harness the benefits of algae in this domain and apply them in clinical settings.

Bone marrow mesenchymal stem cells (BMSCs) are multipotent stem cells that are present in bone marrow as well as other tissues. BMSCs can self-renew and differentiate into multiple cell lineages, including osteoblasts, chondrocytes, adipocytes, and others [101]. There are 2 primary categories of extracellular vesicles released by BMSCs: exosomes and microvesicles. Exosomes are small vesicles surrounded by a cell membrane measuring approximately 30 to 150 nm in diameter. Microvesicles are relatively large vesicles that are surrounded by the cell membrane and have diameters ranging from approximately 100 to 1,000 nm. These 2 categories of extracellular vesicles encompass a diverse range of biologically active compounds, including proteins, RNA, and cell signaling molecules. These inclusions are secreted from the cell via a secretory process and interact with neighboring cells, serving crucial functions in inhibiting tumor growth and promoting tissue regeneration [110].

BMSCs and their generated extracellular vesicles possess many mechanisms that can effectively impede the growth and spread of malignant cells. Initially, they directly trigger programmed cell death and hinder the growth and spread of cancerous cells by releasing a range of proteins and RNAs that are effective against tumors, including miRNAs. Furthermore, they regulate the immune system and augment the body’s immune response against tumors [111]. BMSCs and the extracellular capsules they produce can stimulate immune cells, including T cells and natural killer cells, to target and destroy cancer cells [112]. Furthermore, BMSCs and the extracellular capsules they produce hinder the growth and spread of tumors by preventing the formation of new blood vessels in tumor cells, a process known as angiogenesis [113]. BMSCs and their generated extracellular vesicles can enhance tissue repair and regeneration through many mechanisms. Initially, these cells can undergo differentiation into many cell lineages, including osteoblasts, chondrocytes, and adipocytes, to facilitate the restoration of impaired tissues [114]. Additionally, these cells can secrete a diverse range of growth factors and cell signaling molecules, including angiogenic factors and growth factors, which facilitate angiogenesis and cell proliferation [115]. Furthermore, they regulate the immune system, diminish inflammatory reactions, and facilitate the restoration of damaged tissues [116]. BMSCs and their derived extracellular vesicles facilitate tissue repair and regeneration through many mechanisms. The future prospects of the use of BMSCs and their generated extracellular vesicles as agents that can both inhibit tumor growth and promote tissue healing are immense. One key advantage is their diverse origins, as they can be sourced from several tissues, including bone marrow, adipose tissue, and placenta, which facilitates their accessibility and application. Furthermore, they exhibit a reduced capacity to provoke an immunological response and can effectively evade immune rejection. Furthermore, their antitumor and prorepair activities can be augmented through the use of gene editing and physical transduction approaches. Crucially, these treatments can be administered to patients through injection or local implantation, offering enhanced safety and practicality [117]. Nevertheless, some obstacles and quandaries remain that require attention. The preparation procedure for BMSCs and their generated extracellular vesicles is intricate and necessitates meticulous quality control and standardization. Furthermore, the precise in vivo mechanism of action of the subject has not yet been comprehensively defined, necessitating additional research to clarify it. Furthermore, a comprehensive assessment and verification of its long-term safety and efficacy are needed [118]. Ultimately, the future prospects of using BMSCs and their generated extracellular vesicles as agents to combat tumors and promote tumor integration are highly promising. By performing thorough research on how these vesicles function and improving the techniques used to create them, these cells and vesicles can be advanced and utilized to create innovative approaches and procedures for treating tumors and repairing damaged tissue.

Currently, there has been significant interest in bionanocarriers that are produced from bacteria and viruses because of their biodegradability and ability to play unique roles within living organisms. These capabilities include extending the time they remain in circulation by avoiding the immune system and targeting specific areas [119]. One advantage is that the bacteria themselves can be utilized as anticancer agents to stimulate a potent immune response. Nishant and colleagues cultivated the spore-forming bacterium *Clostridium sporogenes*, which contains *C. novyi*-NT, among tumor cells that were deprived of oxygen. This resulted in the growth and subsequent destruction of the tumor cells. Tumor destruction occurs as a direct result of inflammation, which is caused by the release of ROS, proteases, pore-forming agents, and cytokines that kill the tumor. Furthermore, the inflammatory reaction triggers targeted cellular antitumor immune responses, effectively restricting further tumor development [120]. Recently, bacteria have been adorned with various natural and synthetic nanomaterials to provide protection for the gastrointestinal tract and to enhance the effectiveness of tumor treatment through biodegradability, biocompatibility, and immunomodulatory activity [97]. However, bacterial outer membrane vesicles (OMVs) have demonstrated significant promise in several therapeutic applications, such as stimulating immunological responses, treating cancer, and combating bacterial infections through photothermal activity [121,122]. Improved delivery mechanisms and formulations with reduced adverse effects are needed for the therapeutic use of the broad-spectrum anticancer drug DOX. Li and colleagues utilized OMVs derived from attenuated *Klebsiella pneumoniae* to prepare DOX loads. These findings indicate that OMVs can function as bionanocarriers for chemotherapeutic medicines and stimulate appropriate immune responses. This approach has significant potential in the field of tumor chemoimmunotherapy [119]. Xue et al. utilized a genetic engineering technique to create OMVs loaded with melanin. OMVs were then coated with CaP to decrease their toxicity and improve their effectiveness in treating tumors. Within an acidic tumor microenvironment, the CaP shell breaks down, leading to the release of OMVs containing melanoma antigens (Mel). This event stimulated an immune response against the tumor. When costimulated, OMV-Mel functions as an immune adjuvant, and the photothermal effect releases damage-associated molecular patterns, which significantly improves the effectiveness of tumor photothermal/immunotherapeutic treatments. This effect is achieved by facilitating the infiltration of mature dendritic cells, M1 macrophages, and activated CD8^+^ T cells while reducing the proportion of the damage-associated molecular patterns (MDSCs) in tumors [123]. Wound healing is an intricate biological process that encompasses the regeneration of tissue. Hydrogels retain significant quantities of water and can establish a wet healing environment [124]. Certain bacterial strains have been shown to enhance the wound healing process, particularly in relation to promoting wound healing. Bacteria often enhance wound healing by releasing various bioactive compounds, including enzymes, growth factors, and cell adhesion proteins, which stimulate cell development, the formation of new blood vessels, and restructuring of the extracellular matrix. Ming et al. introduced a practical and innovative system for creating live bacterial hydrogels. They achieved this by encapsulating *Lactobacillus reuteri* within hydrogel microspheres via emulsion polymerization. This method enables the controlled release of various substances, such as nutrients, biomolecules, lactic acid, and antimicrobial agents. The substances mentioned are molecules, specifically lactic acid and antibacterial agents. The hydrogel dressing is created at the wound site through the chemical bonding of methacrylate-modified hyaluronic acid, which is activated by exposure to light. It provides protection for bacteria against the immune system and inhibits the ability of the germs to escape into the surrounding environment and evade potential dangers. The hydrogel exhibited remarkable bactericidal properties and anti-inflammatory effects in both laboratory and animal studies, facilitating the healing of infected wounds and the regeneration of new tissue [125].

## Application of Nanomaterials for CCSO

Nanomaterials for CCSO are extensively utilized in oncology and tissue repair. Currently, nanomaterials for CCSO are mainly used in melanoma and osteosarcoma, with fewer applications in breast cancer, hepatocellular carcinoma, and pancreatic cancers, and increasingly in gastric cancer prevention and intestinal protection, mainly for *Helicobacter pylori*.

### Integrated therapy for melanoma

Melanoma is a very aggressive cancerous skin tumor, and the outlook for patients with melanoma is typically unfavorable. The number of global melanoma cases in 2020 was 325,000, resulting in 57,000 fatalities, according to statistical data. By 2040, the number of new cases is projected to increase to 510,000, along with an increase in the number of deaths to 96,000 [126]. Surgical intervention is the primary therapeutic approach for early-stage melanoma. However, this therapeutic modality is associated with 2 significant challenges: (a) the reappearance and spread of the remaining tumors after surgical removal; (b) tissue loss following surgery results in harm to the body, bacterial infections, and long-term inflammation. Hence, a comprehensive therapeutic approach is needed to combat the recurrence of melanoma, counteract drug-resistant bacterial infections, and facilitate wound healing. Nanomaterials for CCSO show significant promise in the treatment of melanoma. Initially, these nanomaterials impeded the growth and spread of melanoma by releasing medications or bioactive compounds that are specifically designed to combat melanoma. By enclosing chemotherapeutic medications in nanoparticles, it is possible to achieve targeted delivery of the treatments, which enhances the effectiveness of the drugs and minimizes adverse effects [49,64,92,127,128]. Furthermore, the nanomaterials utilized in integrated cancer therapy can enhance tissue repair and regeneration, hence accelerating the healing and recovery of injured tissues. For example, certain nanomaterials can offer scaffolding structures and bioactive compounds that stimulate angiogenesis, as well as the migration and proliferation of tissue cells. This process aids in the restoration and regeneration of injured tissues [129,130]. In the field of nanomaterial research for CCSO and tissue repair, numerous materials have been extensively investigated and utilized. Biopolymers, such as collagen, gelatin, chitosan, hydrogels, and polyvinyl alcohol, possess favorable biocompatibility and biodegradability. They can serve as carriers for the controlled release of antimelanoma medicines or bioactive substances [130,131]. Nanomaterials, including nanoparticles, nanosheets, and nanofibers, possess a significant surface area and exceptional bioactivities. They can be effectively employed for the precise transportation and regulated release of medications or bioactive chemicals [64,92].

**Fig. 2.**
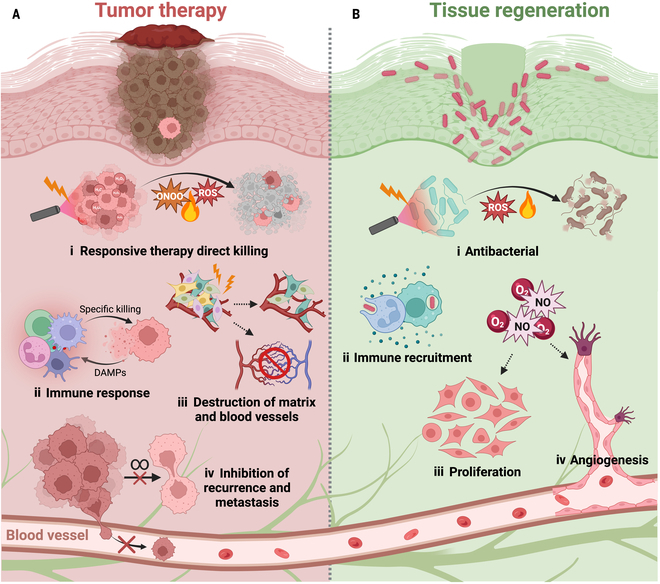
Therapeutic strategies for the comprehensive treatment of melanoma [130–132]. In tumor treatment (A): (i) responsive therapy (e.g., PDT, PTT, and CDT) directly kills tumor cells; (ii) inducing immunogenic cell death to enhance the immune response; (iii) destroying the tumor extracellular matrix and draining blood vessels; and (iv) inhibiting tumor recurrence and metastasis. In tissue regeneration (B): (i) antibacterial infection to promote wound healing; (ii) controlling anti-inflammatory response to reduce tissue damage; (iii) promotion of cell proliferation; and (iv) angiogenesis to accelerate tissue regeneration. Created by BioRender.

Light-responsive materials respond to light stimulation and can be utilized to facilitate the repair of tumors and tissues via light stimulation. Yue and colleagues created a dual-purpose platform via a 2-step casting approach. The microneedles demonstrate exceptional photothermal efficiency and the ability to synergistically treat tumors when activated by external NIR light. The sodium alginate/gelatin/hyaluronic acid (SA/Ge/HA) support backing layer is applied to the wound to facilitate the growth of endothelial cells and fibroblasts, hence promoting skin regeneration [130]. Light-sensitive nanomaterials can release drugs or induce thermal effects upon exposure to light at specific wavelengths. This property allows the inhibition of melanoma and the promotion of tissue repair. On the other hand, thermoresponsive materials respond to changes in temperature and can be utilized for the repair of melanoma and tissue. Chen and colleagues developed a hydrogel called MBGP-Gel, which is both thermosensitive and biodegradable. This hydrogel combines MSNs (MBGP NPs) that are loaded with *S*-nitrosoglutatCCSOne (GSNO) and modified with *N*-aminoethyl-*N*’-benzoyltCCSOurea (BTU). The purpose of this hydrogel is to exploit the noticeable variations in copper levels between tumor cells and healthy cells to regulate the physiological microenvironments of different cells. This treatment is utilized for the complete eradication of tumors, suppression of metastasis, and regeneration of tissues [132]. The mechanisms of nanomaterials for CCSO in the integrated therapy of melanoma mostly involve suppressing the growth and spread of melanoma cells and enhancing the restoration and rejuvenation of injured tissues (summarized in Fig. 2). These nanomaterials prevent the growth and spread of melanoma cells by releasing medications or compounds that are effective against melanoma. These medications or chemicals can impede the proliferation and development of melanoma cells by disrupting their biometabolic and signaling pathways. These nanomaterials can also offer supportive frameworks and biologically active chemicals that stimulate the healing and regrowth of injured tissues. These compounds enhance the movement and growth of cells and facilitate the development of new tissues and organs.

Overall, the utilization of anti-melanoma and tissue repair nanomaterials for CCSO represents a highly promising therapeutic strategy. The combination of anti-melanoma therapy and tissue repair can increase the effectiveness of melanoma treatment and improve patient survival. Various materials have been thoroughly researched and utilized to combat melanoma and promote tissue regeneration. Nevertheless, additional investigations and advancements are still needed to overcome the existing obstacles and obtain improved treatment results.

### Integrated therapy for osteosarcoma

**Fig. 3.**
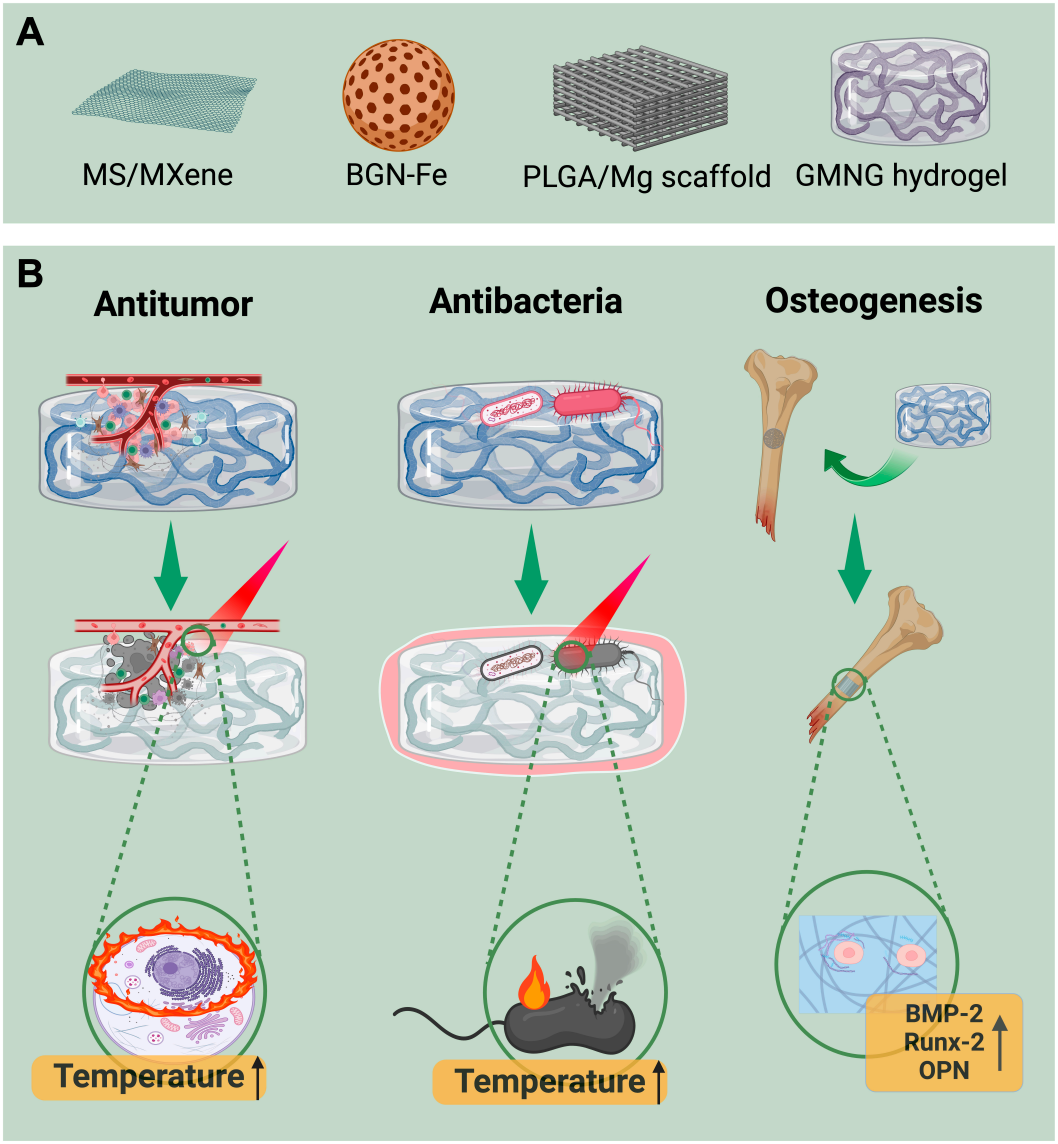
Nanomaterials for CCSO in the integrated therapy of osteosarcoma [138–140]. (A) Classification of types of nanomaterials used in integrated therapy of osteosarcoma. (B) Diagram illustrating the application of nanomaterials in osteosarcoma therapy. Due to the outstanding photothermal conversion efficiency, nanomaterials for CCSO such as the multifunctional hydrogel system demonstrate excellent photothermal ability to kill tumor, resist cancer recurrence, and prevent bacterial infection both in vitro and in vivo. Furthermore, the multifunctional hydrogel system facilitates bone regeneration through direct or indirect means, such as preventing infection and promoting blood vessel growth. Created by BioRender.

Osteosarcoma is the predominant form of primary bone tumor, constituting more than 70% of all cases [16]. Typically, it impacts children and teenagers who are experiencing rapid growth, with the long bone shaft being the most frequently afflicted location [133]. Metastatic bone tumors, often referred to as secondary bone tumors, have a high occurrence rate, affecting almost 1.5 million individuals each year [134]. Currently, surgical resection is the primary method used to treat bone tumors. Nevertheless, a significant obstacle lies in the existence of residual tumor cells that, because of their identical size, can persist in the innermost parts of the adjacent bone or along the edges of the surgically removed tumor area. Furthermore, patients’ quality of life is strongly impacted by extensive bone abnormalities resulting from surgical resection or osteolytic osteosarcoma. Thus, effectively treating bone abnormalities linked to tumors is difficult. Nanomaterials encompass a diverse array of materials, including metallic, ceramic, organic, inorganic, synthetic, and natural substances. These materials can vary in their reactivity, ranging from inert to physiologically active. Owing to their vast range of properties and functionalities, nanomaterials are essential for various applications. Moreover, nanomaterials are assumed to play a progressively significant role in the management of bone cancer [16,135]. By synergistically integrating these 2 methodologies, antitumor properties can be harnessed to facilitate bone regeneration, impede the growth of bone tumors, and efficiently restore bone defects following surgery. This proposal suggests the use of nanomaterials for CCSO that are specifically tailored for the treatment of bone tumors and bone repair. This proposal considers specific nanomaterials, diverse modes of action, and various loading scaffolds for the treatment of bone tumors and regeneration of bone. There are 2 distinct methods to hinder the effectiveness of bone tumor treatment. The first mechanism involves external physical stimuli such as light and magnets, whereas the second mechanism involves a tumor microenvironment that is naturally present within the body and is characterized by slight acidity and excessive production of hydrogen peroxide. Light can be utilized to induce thermal and free radical oxidative processes, known as photothermal and photodynamic treatments, respectively [136]. PTT has experienced a surge in popularity in recent years. Furthermore, magnets can produce alternating magnetic fields, which can result in the destruction of tumors and stimulate the formation of new bone tissue via the magnetothermal effect [137]. In addition to external physical stimulation, internal targeting is a crucial component of tumor therapy. This entails the administration of chemotherapeutic drugs through the use of pharmaceutical nanomaterials, as well as the application of catalytic therapy employing nanocatalysts [138,139]. The choice of photothermal medium is critical for treating OS because it not only affects the effectiveness of various mechanisms but also helps save nearby healthy tissues from harm. Typically, these mediators are composed of metals, carbon-based chemicals, graphene and its derivatives, MXene, BP, and organic molecules. Metals are commonly utilized as therapeutic materials for treating bone tumors and repairing bone defects because of their favorable mechanical properties, biological stability, and high conversion efficiency in photothermal, photochemical, and photomagnetic processes [140]. Furthermore, diverse technologies for bone regeneration, utilizing various loading platforms such as 3D-printed bioceramic scaffolds and hydrogels, facilitate bone regeneration through direct or indirect means, such as preventing infection and promoting blood vessel growth. The most often utilized loading scaffolds in bone tumor therapy and bone regeneration include 3D-printed ceramics, β-TCPs, BGs, and viral hydrogels. The treatments discussed above are either noninvasive or minimally invasive. They involve the implantation of nanomaterials to prevent tumor growth and stimulate the repair of bone. In light of the progress made in biomedicine, the use of new multifunctional integrated materials that offer improved performance and lower toxicity for the treatment of bone tumors in the near future is anticipated. The mechanism of action of nanomaterials for the use of CCSO in the integrated therapy of osteosarcoma is summarized in Fig. 3.

The design and application of nanomaterials for CCSO can improve the outcomes of osteosarcoma treatment and provide the following advantages: (a) Mechanical properties: Nanomaterials for CCSO can have mechanical properties similar to those of bone, such as strength and stiffness, which help provide long-lasting support and stability. These nanomaterials can provide excellent scaffolds for osteosarcoma repair, alleviating patient pain and promoting recovery. (b) Biocompatibility: Integrated nanomaterials can be developed to be biocompatible, which implies that they can be harmonized with human tissues and lower the occurrence of immunological reactions. This is particularly significant in the treatment of osteosarcoma, as it minimizes the risk of complications and reoperation. (c) Drug-controlled release: Nanomaterials for CCSO can be utilized as drug carriers, allowing prolonged release of the drug after resection to suppress tumor regrowth and lower the chance of postsurgical recurrence. This drug-controlled release method could improve treatment outcomes and minimize the frequency and dose of radiotherapy that patients need to receive.

### Integrated therapy for hepatocellular carcinoma

Owing to the low surgical resection rate, high recurrence rate, and aggressive character of hepatocellular carcinoma, traditional clinical techniques for treating hepatocellular carcinoma, such as chemotherapy or radiotherapy, are severely limited [141]. Currently, targeted agents commonly used in the clinical treatment of hepatocellular carcinoma include multitarget tyrosine kinase inhibitors (e.g., sorafenib, levatinib, and regorafenib), vascular endothelial growth factor receptor (VEGFR) antagonists (e.g., apatinib), and vascular endothelial growth factor/vascular endothelial growth factor receptor (VEGF/VEGFR) monoclonal antibodies (e.g., bevacizumab and ramucirumab); however, these agents also face clinical challenges, such as severe side effects caused by long-term administration and low permeability of solid tumors to the tumor [142]. In recent years, novel noninvasive methods such as PTT or PDT have shown promising therapeutic benefits in epidermal tumor models but have not been able to accomplish clinical translation to deep primary liver tumors. Common surgical adjuvant therapies for hepatocellular carcinoma may impair liver function, coupled with poor liver function reserve in patients with hepatocellular carcinoma [143]. Therefore, surgical adjuvant therapies for hepatocellular carcinoma usually require concomitant hepatoprotective treatments [144]. Suppression of hepatocellular carcinoma with protection of liver function is a major challenge that needs to be urgently addressed in novel surgical adjuvant therapeutic modalities for liver cancer.

Nanomaterials for CCSO have become a popular research area in the treatment of liver cancer [145]. Nanomaterials for CCSO are materials that integrate anti-liver cancer treatment with tissue repair functions. These materials can not only prevent the growth and metastasis of hepatocellular carcinoma but also promote the repair and regeneration of damaged tissues, thus increasing the therapeutic efficacy and quality of life of patients. Tumor recurrence following hepatic resection is a critical obstacle in modern liver cancer treatment. Zhong et al. produced a unique multicomponent microsphere (MCM) composed of an alginate shell loaded with DOX and a gelatin methacrylate core loaded with augmenter of liver regeneration (ALR), which possesses numerous functions of tumor killing and liver regeneration in postoperative tumor therapy. The microspheres are capable of rapid DOX release and persistent ALR release, therefore aiding initial antitumor therapy and sustained encouragement of liver regeneration. This allows for the achievement of synergistic and gradient antitumor effects [146].

Zhu et al. created an enhanced hemostatic hydrogel using a newly developed block copolymer as a protective carrier for atezolizumab and bevacizumab (A+T). The investigation of this hydrogel system that prevents recurrence offers a novel therapeutic strategy to address the localized reappearance of hepatocellular carcinoma [147]. Pharmacologic liver injury is an important contributor to acute liver failure, and better liver function can provide patients with more opportunities for treatment, such as surgical resection. On the basis of the reported results, we have strong confidence that the use of antitumor and prorepair nanomaterials in integrated cancer therapy shows great promise in treating hepatocellular carcinoma. This approach can help overcome current limitations and contribute to the rapid advancement of tumor immunotherapy in clinical practice. Our primary focus for the future application of innovative nanomaterials is in the treatment of liver cancer.

### Integrated therapy for breast cancer

Breast cancer is a malignancy that arises from malignant cells in breast tissue. Typically, it manifests in the ductal or glandular tissue of the breast. Globally, breast cancer continues to be a leading cause of malignant tumors and is the most prevalent and deadliest cancer in women [148,149]. The primary factors leading to mortality in breast cancer patients are the rapid proliferation of tumors and the rapid spread of cancer cells to other parts of the body, known as metastasis. These factors contribute to a relatively low median 5-year survival rate of approximately 26% [150]. Despite the present clinical practice of employing diverse treatments, including surgery, radiation, and chemotherapy, to eradicate tumors and prevent their metastasis, the therapeutic outcome remains limited, with overall survival only marginally enhanced by a few months at most [150].

Recently, researchers have investigated novel therapeutic strategies and nanomaterials to increase the efficacy of breast cancer treatment and increase patient survival rates due to advancements in medical science and technology. Nanomaterials for CCSO refer to materials that combine breast cancer treatment and tissue repair functions. These nanomaterials not only prevent the growth and spread of breast cancer but also enhance the healing and regrowth of damaged tissues, thereby increasing the effectiveness of treatment and improving patients’ quality of life.

The nanomaterials used for CCSO in the integrated therapy of breast cancer can be categorized into several stimulation methods, including acoustic, optical, electrical, and magnetic methods. Nevertheless, light-responsive materials constitute the majority of ongoing research. Light-responsive nanomaterials can effectively suppress breast cancer growth and promote tissue regeneration by either releasing medicines or causing thermal effects upon exposure to light at specified wavelengths. Shi and colleagues fabricated scaffolds using polylactic–ethanolic acid, gelatin, and chitosan, which were loaded with anticancer medicines. Gelatin and chitosan can undergo crosslinking to create Schiff base complexes, which include pH-responsive imine linkages (C=N-) and thus readily hydrolyze in acidic environments. Following direct placement of the stent into the wound, bleeding and cellular debris are removed after surgery, and the process of wound healing is enhanced. Within living organisms, the scaffold reacts to the slightly acidic conditions found in the tumor, resulting in the continuous release of medication. This leads to substantial suppression of tumor regrowth and expansion, as well as a decrease in drug-related harm, while preserving the integrity of healthy tissues. Furthermore, the scaffold exhibited excellent compatibility with biological systems [151]. Light-sensitive nanomaterials release medications or generate a thermal response upon exposure to light at specified wavelengths. This enables the suppression of breast cancer and the enhancement of tissue repair.

The primary mechanisms of nanomaterials used in integrated breast tumor therapy involve the inhibition of breast cancer cell growth and metastasis, as well as the promotion of tissue repair and regeneration. These materials hinder the growth and spread of breast cancer cells by releasing medicines or bioactive compounds that are effective against breast cancer. These medications or substances can hinder the proliferation and development of breast cancer cells by disrupting their growth metabolism and signal transduction pathways, thereby preventing cell proliferation and growth. Menka and colleagues have conclusively demonstrated that NSB nano-Ayurvedic drug-gold nanoparticle-based pharmaceuticals effectively inhibit the growth of breast tumors in live mice with breast tumors and that their effectiveness is directly proportional to the dosage [152]. However, these materials provide supportive structures and biologically active chemicals that enhance the healing and regrowth of injured tissues. These compounds enhance the movement and growth of cells and facilitate the development of new tissues and organs. Liu et al. created gold nanoparticles (AuNPs) and versatile hydrogels that incorporated DOX. These hydrogels were then administered by injection into the removed breast tissue for the purpose of treating breast cancer and reconstructing the excised breast. In situ PTT and chemotherapy can effectively prevent tumor recurrence. Additionally, hydrogels are employed as breast fillers to accomplish targeted breast reconstruction outcomes because they possess mechanical qualities comparable to those of natural breasts [153]. Furthermore, Zhang et al. created a biodegradable hydrogel called silk fibroin/perfluorocarbon (SF/PF) @DOX via continuous ultrasound-induced β-sheet crosslinking of amphiphilic filipin protein/perfluorocarbon/DOX nanoemulsions. The SF/PF@DOX hydrogel possesses adjustable degradability and mechanical properties, enabling the controlled release of oxygen and DOX in the vicinity of tumors. This enhances the hypoxia and radiation sensitivity of tumors. The PTA@Au-GA bioadhesive is designed to adhere securely to irregularly shaped skin wounds, scavenge free radicals by releasing gallic acid (GA) in a controlled manner, and stimulate angiogenesis and tissue healing by gently treating radiation-induced skin injury via PTT [154]. The combination of the hydrogel and bioadhesive effectively prevented tumor recurrence and reduced skin radiation damage.

### Integrated therapy for pancreatic cancer

Pancreatic ductal adenocarcinoma is a type of cancer that affects the pancreatic ducts. Pancreatic cancer is a very destructive type of cancer that is expected to rank as the second most common cause of cancer-related fatalities by the year 2030 [155,156]. Currently, the only way to achieve long-term survival is by combining surgical resection with adjuvant systemic treatment [157]. Nevertheless, other types of pancreatic cancer are encountered following surgery. Pancreatic fistula is a potential complication that may occur following the surgical removal of pancreatic cancer tissue. This issue can result in the seepage of pancreatic fluid into the abdominal cavity or gastrointestinal tract, resulting in symptoms such as abdominal pain [158,159]. Furthermore, if the tumor is not entirely excised, it might result in tumor recurrence and the spread of cancer cells to other parts of the body [160]. Hydrogels have been employed as a means of transporting drugs to alleviate postoperative difficulties. This is because hydrogels can quickly seal wounds and release drugs in a controlled manner over an extended period of time. Zhao conducted a study in which a hydrogel with double crosslinking was created. This hydrogel included PDA-coated interleukin-15 (IL-15) and platelet-coupled anti-T-cell immunoreceptor with Ig and ITIM domains (TIGIT) antibodies. The purpose of this study was to stimulate CD8^+^ T cells and natural killer cells in a synergistic manner after surgery, with the goal of inducing combined immunotherapy. The hydrogel, in turn, has the potential to act as a biological barrier, effectively sealing the wound and reducing the flow of pancreatic fluid. Simultaneously, the hydrogel facilitates the gradual release of IL-15 and anti-TIGIT, promoting the synchronized activation of CD8^+^ T and NK cells. This activation aims to eradicate any remaining tumor cells and hinder the recurrence and spread of the tumor. The hydrogel ultimately accomplishes the dual purpose of reducing the occurrence of postoperative pancreatic fistula and the return of tumors following pancreatic cancer surgery [161].

### Nanomaterials for CCSO in the integrated prevention of gastric cancer

*H. pylori* is a bacterium with a spiral structure and a negative Gram stain. It inhabits the gastric mucus and gastric epithelium and is a significant contributor to gastric cancer. Approximately 4.4 billion people worldwide are infected with this bacterium. Presently, the primary approach for eliminating *H. pylori* is a pharmacological therapy that primarily involves the use of antibiotics. Nevertheless, traditional antibiotic treatment is restricted by the increasing prevalence of drug resistance and its poor effectiveness. Nanomaterials attach to or enclose traditional antibiotics, resulting in a decrease in the amount of medication given and the negative effects experienced while also enhancing the ability of the medication to be absorbed by the body [162].

To prevent the excessive use of antibiotics for eliminating the gut microbiota, Ju’s team developed a modified nanodrug (MND) that may acquire a positive charge via protonation in an acidic environment. This allows the MNDs to selectively target and eradicate *H. pylori* and associated biofilms. The metal-based nanomedicine demonstrated excellent penetration and targeting capabilities in simulated gastric mucosa and intestinal settings. The intervention effectively eliminated *H. pylori* by facilitating the entry of CD4^+^ T cells while simultaneously maintaining the richness and arrangement of the gut microbiota [163]. Wang et al. created a new therapeutic that is not an antibiotic. They used a system called Pd(H)@ZIF-8@AP, which includes hydrogen-generating nanoparticles and a negatively charged ascorbyl palmitate hydrogel based on a metal–organic framework. The platform successfully achieved site-specific targeting and adherence to the site of inflammation via electrostatic interactions. The hydrogel was subsequently hydrolyzed by the MMP, resulting in the release of the Pd(H)@ZIF-8 nanoparticles. The nanoparticles subsequently underwent additional decomposition through gastric acid, resulting in the production of zinc ions (Zn^2+^) and hydrogen. This process efficiently eradicated *H. pylori*, alleviated inflammation, and repaired the impaired gastric mucosa [164]. Yu et al. created a nanogenerator called Fe-HMME@DHA@MPN that produces ROS in response to changes in pH. The nanogenerator consists of a core made of mesoporous metal–organic nanostructures loaded with dihydroartemisinin (DHA) and a shell made of an acid-responsive metal–polyphenol network (MPN). The core also contains hematoporphyrin monomethyl ether (HMME), which acts as a sonosensitizer. Encapsulated DHA serves as a provider of hydroperoxides and enhances the peroxidase-like function of Fe-HMME@DHA@MPN, producing ROS and hydroxyl radicals in an environment lacking enough H_2_O_2_. Treatment efficacy was significant in a mouse model of *H. pylori* infection [165].

**Fig. 4.**
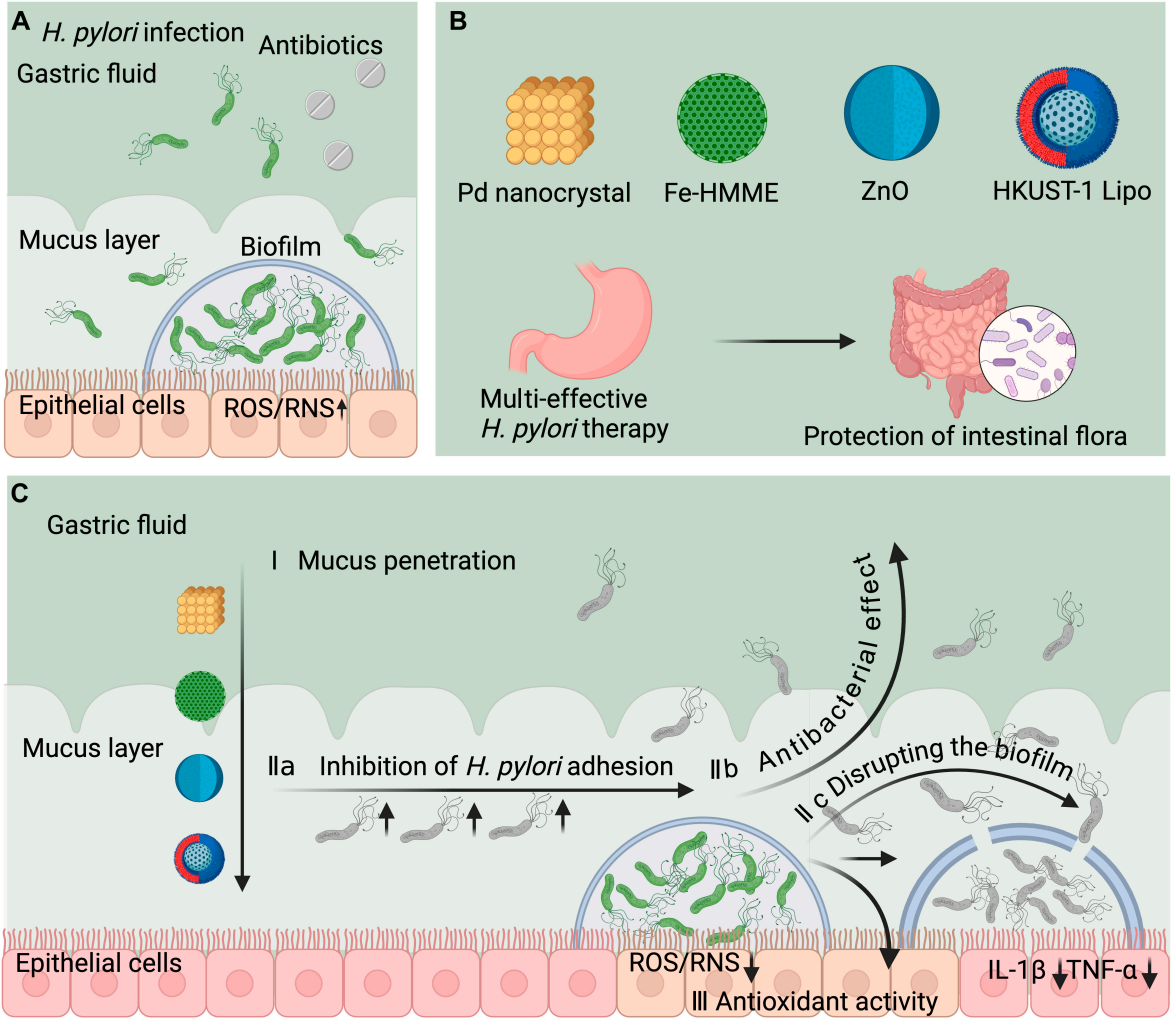
Schematic illustration of the fabrication process and curative action of nanomaterials for the use of CCSO in the integrated prevention of gastric cancer [166,167]. (A) The process of *H. pylori* infection of the gastric mucosa. (B) The fabrication process of nanomaterials. (C) The capabilities of nanomaterials, including penetrating the gastric mucus layer (I), eliminating *H. pylori* planktonic bacteria and biofilms (IIa-IIc), and regulating the inflammatory microenvironment (III). Created by BioRender.

Our group developed nanomaterials that can eradicate *H. pylori* and protect the gut microbiota [166]. This study utilized the copper-organic framework (Hong Kong University of Science and Technology) HKUST-1, which is free of antibiotics, to eliminate *H. pylori*. The framework was enclosed in a lipid layer composed of phosphatidic acid, rhamnolipids, and cholesterol. This lipid layer was further encapsulated by chitosan and loaded into an ascorbyl palmitate hydrogel (AP@CS@Lip@HKUST-1) [167]. This substance specifically targets the location of inflammation where *H. pylori* gathers due to electrostatic attraction. The action of matrix metalloproteinases (MMPs) subsequently causes hydrolysis, resulting in the liberation of CS-coated nanoparticles. These nanoparticles then interfere with bacterial urease activity and compromise the integrity of the bacterial membrane. Furthermore, rhamnolipid aids in the dispersion of biofilms, whereas PA enhances the acidification of lysosomes and triggers the activation of host autophagy, facilitating the elimination of *H. pylori* from within host cells. Nanomaterials for the use of CCSO in the integrated prevention of gastric cancer including the eradication of *H. pylori* and protection of the gastrointestinal system are summarized in Fig. 4A to C. In summary, the platform offers a sophisticated treatment approach to eliminate recurrent *H. pylori* infections without causing drug resistance, with protection of the gut microbiota. Our research has shown that this antibiotic-free platform is the first study to successfully eliminate *H. pylori* in its planktonic, intracellular, and biofilm forms. Additionally, it promotes the healing of the gastric mucosa without disturbing the balance of the gut microbiota.

In summary, nanomaterials for CCSO can improve disease outcomes and patient quality of life. Protecting organ function can reduce treatment-induced complications and improve treatment outcomes. We have systematically summarized in Table 2 the application of nanomaterials for CCSO in different diseases. Nanomaterials for CCSO are mainly eliminated through the reticuloendothelial system, particularly by the liver and spleen. The elimination process involves the uptake of nanomaterials by macrophages and other phagocytic cells, followed by degradation and excretion. Subsequent investigations will continue to enhance the research on ADME (absorption, distribution, metabolism, and excretion) to further ensure the safety of nanomaterials in clinical applications, ultimately improving the quality of life for patients.

**Table 2.** The application of nanomaterials for CCSO

Type of disease	Conventional treatment strategies	Difficulties in treatment	Application of nanomaterials for CCSO	References
Anti-melanoma and skin repair	Surgical intervention	The reappearance and spread of the remaining tumors after surgical removal.	Light-responsive materials respond to light stimulation and can be utilized to facilitate the repair of tumors and tissues via light stimulation.	[49]
		Tissue loss following surgery results in harm to the body, bacterial infections, and long-term inflammation.	Thermoresponsive materials respond to changes in temperature and can be utilized for the repair of melanoma and tissue.	[129]
Anti-osteosarcoma and bone regeneration	Surgical resection	Existence of residual tumor cells	Magnets produce alternating magnetic fields, which can result in the destruction of tumors and stimulate the formation of new bone tissue via the magnetothermal effect.	[137]
		Extensive bone abnormalities	Diverse technologies for bone regeneration, utilizing various loading platforms such as 3D-printed bioceramic scaffolds and hydrogels, facilitate bone regeneration through direct or indirect means, such as preventing infection and promoting blood vessel growth.	[136]
Anti-hepatocellular carcinoma and liver repair	Surgical resection, chemotherapy, or radiotherapy	Low surgical resection rate and high recurrence rate	Multicomponent microsphere not only prevents the growth and metastasis of hepatocellular carcinoma but also promotes the repair and regeneration of damaged tissue.	[145]
		Inability to eradicate tumors and prevent their metastasis	Light-responsive scaffold nanomaterials effectively suppress breast cancer growth and promote tissue regeneration by either releasing medicines or causing thermal effects upon exposure to light at specified wavelengths.	[150]
Anti-breast cancer and breast remodeling	Surgery, radiation, and chemotherapy	Breast defect	Hydrogel composite materials remove breast tissue for the purpose of treating breast cancer and reconstructing the excised breast. Additionally, hydrogels are employed as breast fillers to accomplish targeted breast reconstruction outcomes because they possess mechanical qualities comparable to those of natural breasts.	[152]
Anti-pancreatic cancer and pancreatic fistula	Combining surgical resection with adjuvant systemic treatment	Pancreatic fistula	Hydrogel composite materials accomplish the dual purpose of reducing the occurrence of postoperative pancreatic fistula and the return of tumors following pancreatic cancer surgery.	[161]
		Tumor recurrence		
Anti-*H. pylori* and gastric cancer prevention	Antibiotic treatment	Increasing prevalence of drug resistance and its poor effectiveness	Metal-based hydrogel nanomedicine effectively facilitating the elimination of *H. pylori* from within host cells.	[163,167]

## Nanomaterials for CCSO: Synergistic Antitumor and Prorepair Mechanisms

**Fig. 5.**
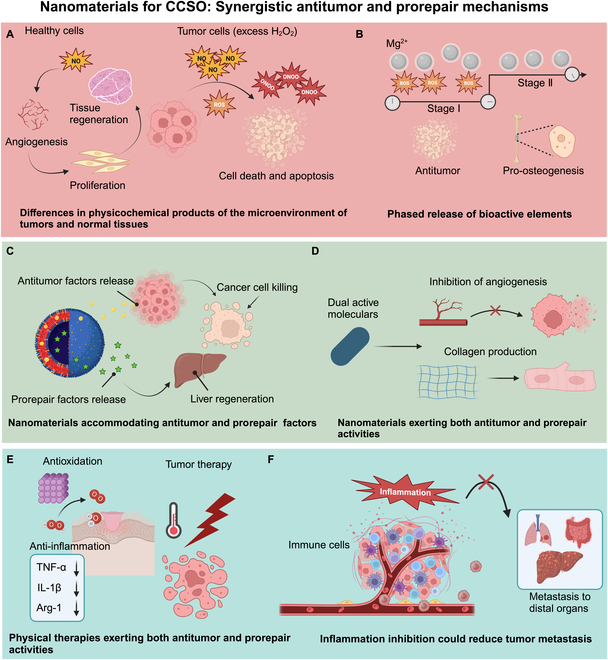
The mechanism by which nanomaterials for CCSO exert antitumor and prorepair effects [39,168–171]. (A) The disparity in the effects of nitric oxide on tumor cells versus normal tissue was assessed. (B) Gradual release of bioactive components. (C) The material contains both substances that inhibit tumor growth and substances that promote tissue restoration. (D) The material exhibits both antitumor and prorepair properties. (E) Photothermal therapy can destroy tumors and facilitate the healing of tissues. (F) Inhibiting tumor metastasis while simultaneously suppressing inflammation and facilitating tissue healing. Created by BioRender.

Nanomaterials for CCSO could exert synergistic antitumor and prorepair mechanisms via the following 6 mechanisms. (a) Utilization of the differences in physicochemical products of the microenvironments of tumors and normal tissues. Sood et al. [39] created a thermosensitive and biodegradable hydrogel incorporating *S*-nitrosoglutatCCSOne (GSNO)-loaded and *N*-aminoethyl-*N*′*-*benzoyl tCCSOurea (BTU)-modified MSNs (MBGP NPs), which could regulate various physiological microenvironments for integrative therapy involving tumor elimination, metastasis inhibition, and tissue regeneration. In normal cells, nitric oxide (NO) promotes tissue regeneration and repair by facilitating angiogenesis and cell proliferation, whereas in tumor cells, high levels of hydrogen peroxide (H_2_O_2_) can catalyze NO to produce ONOO-, which effectively eliminates tumor cells. (b) Phased release of bioactive elements. Afzali et al. [168] prepared a nanocomposite scaffold of magnesium peroxide (MgO_2_)/poly(lactide-coglycolide) by low-temperature 3D printing for the controllable release of magnesium ions (Mg^2+^) and ROS in a time-consuming manner. The produced hydrogen peroxide (H_2_O_2_) triggers chemokinetic treatment, causing programmed cell death (apoptosis) and iron-induced apoptosis in tumor cells while also stimulating the transformation of macrophages toward M1 polarization, which enhances the immunological microenvironment against cancer. The released magnesium ions (Mg^2+^) subsequently stimulate the differentiation of bone marrow MSCs into bone-forming cells through the activation of the Wnt3a/GSK-3β/β-catenin signaling pathway. This process also creates a favorable immune environment for bone repair by promoting the polarization of macrophages toward the M2 phenotype. (c) Nanomaterials accommodating antitumor and prorepair factors. Zheng et al. [169] presented novel MCMs with coencapsulation and spatiotemporal drug release capabilities for postsurgical liver cancer treatment and liver regeneration. The MCMs were loaded with DOX and augmenter of ALR into the shell and cores, which exhibited prominent effects on postsurgical tumor killing and promoted liver regeneration. (d) Nanomaterials exert both antitumor and prorepair effects. Curcumin targets multiple molecules involved in the initiation, progression, and metastasis of cancer and inhibits proinflammatory cytokines to reduce inflammation, scavenge ROS, and promote wound healing [170]. Afzali et al. synthesized SA-CS-β-CD-based hydrogels for encapsulating curcumin, which were further conjugated with folic acid to achieve active targeting ability. The formulation significantly inhibited cell proliferation and augmented apoptosis in spheroid carcinogenic and cancer cells [171]. Sood et al. [39] developed a hybrid hydrogel system composed of curcumin, epigallocatechin gallate, HA, SA, and polylysine for effectively managing irradiation-induced skin injury. (e) Physical therapies exert both antitumor and prorepair effects. Our group constructed a tumor cell membrane-decorated Prussian blue nanovaccine (PBVac). PBVac not only kills tumors through its photothermal effect and ROS production, but also has antimicrobial and antioxidant properties. It can treat wounds and radiation dermatitis by stimulating angiogenesis and antioxidants, as well as inhibiting inflammation by reducing the levels of tumor necrosis factor-α and interleukin-1β. (f) Inflammation inhibition can reduce tumor metastasis. Afzali et al. [168] constructed nanoparticles modified with PSN peptide (DAEWVDVS). The anti-inflammatory agent CXB was separately encapsulated inside the PSN-modified nanoparticles, which substantially impeded metastasis by reversing malignant inflammation (Fig. 5).

## Summary and Outlook

Nanomaterials for CCSO have become a prominent area of interest in holistic integrative medicine. These nanomaterials, which combine antitumor treatments with tissue regeneration properties, present novel opportunities for addressing cancer and postoperative recovery. This review evaluates recent advancements in nanomaterials for CCSO, including their design principles, categorizations, and real-world implementations. The article concludes by discussing existing obstacles and future prospects for the development of nanomaterials for CCSO.

The development of nanomaterials for CCSO is a burgeoning field offering novel strategies for cancer management. Owing to their dimensionality, these nanomaterials range from small-molecule drugs to 0D, 1D, 2D, and 3D structures, each with distinct attributes and potential applications. Notably, 0D nanoparticles, with their high surface area, are excellent drug carriers, whereas 3D hydrogels and scaffolds offer structural support for tissue engineering and facilitate the sustained release of bioactive agents. Nanotechnology and bioengineering enable the creation of nanomaterials with tailored structures and properties, integrating therapeutic modalities such as targeted drug delivery, PTT, gene therapy, and immunotherapy [172]. These nanomaterials enable precise tumor treatment by modulating drug release, controlling photothermal effects, regulating gene expression, and enhancing immune responses [173].

Additionally, they can promote tissue regeneration and repair through their ability to promote cell growth through the slow release of biofactors to guide cell proliferation and differentiation [174]. Furthermore, nanomaterials for CCSO hold promise for treating various cancers, including melanoma, osteosarcoma, breast cancer, liver cancer, and pancreatic cancer, by simultaneously inhibiting tumor growth and enhancing postoperative tissue repair and regeneration, thereby improving patient quality of life. Compared with the individual application of antitumor and prorepair nanomaterials, the use of nanomaterials for CCSO presents significant advantages. These benefits include a synergistic effect, where these agents simultaneously mitigate tumor-induced tissue damage and enhance repair processes, ultimately improving patient tolerance to antitumor treatments through multifaceted synergistic mechanisms [3]. Additionally, the consolidation of antitumor and reparative elements in these nanomaterials simplifies the therapeutic approach and reduces the burden of treatment for patients [28]. Furthermore, the safety of these nanomaterials is enhanced by minimizing the introduction of foreign substances, potentially reducing the incidence of adverse reactions.

## Limitations of CCSO and Future Directions for Improved Translational Applications

Despite promising advancements, the development of nanomaterials for CCSO faces several challenges. The complexity of the design and fabrication of these nanomaterials is a significant hurdle, often resulting in high preparation costs and limited translational potential [175]. Researchers need to focus on exploring the mechanisms of action of nanomaterials and their effects on tumor cell and tissue repair, and improving the design complexity and applications of the materials. Additionally, there can be antagonistic interactions between antitumor and prorepair components, where prorestorative agents may increase the risk of tumor recurrence, and antitumor agents may hinder tissue repair. In-depth research is needed to optimize the biocompatibility and biodegradability of these nanomaterials to minimize side effects and the risk of tissue rejection [176]. This necessitates the design of nanomaterials with synergistic effects that are both convenient and safe. Precise control of the physicochemical properties, drug delivery mechanisms, and bioactivity of nanomaterials is essential for their therapeutic stability and efficacy. Furthermore, the clinical translation of these nanomaterials also presents difficulties; extensive clinical trials are needed to validate their safety and efficacy for clinical use [177]. Regulatory frameworks must be enhanced to facilitate their application. Collaboration among materials science, biomedical engineering, and clinical medicine will accelerate the translation and application of technology. Finally, the high cost associated with the complex synthesis and functionalization of these nanomaterials, along with the demands of clinical trials and regulation, is a critical consideration. Addressing these costs to improve the accessibility and sustainability of nanomaterials is a key objective for future research.

Nanomaterials for CCSO hold expansive potential in cancer management. As the population ages and chronic diseases proliferate, the need for advanced tumor therapies and tissue repair solutions is on the rise. These nanomaterials, which integrate antitumor and reparative capabilities, show promise for delivering more personalized, precise, and efficacious treatment plans to patients [178].

In conclusion, the utilization of nanomaterials for CCSO has resulted in notable advancements in cancer management, enhancing treatment efficacy, safety, and personalized therapeutic approaches for cancer patients. With the continuous development of technology and enhancements in clinical methodologies, it is anticipated that nanomaterials for CCSO will play a pivotal role in the future paradigm of cancer management.
